# Impact of Scyphozoan Venoms on Human Health and Current First Aid Options for Stings

**DOI:** 10.3390/toxins10040133

**Published:** 2018-03-23

**Authors:** Alessia Remigante, Roberta Costa, Rossana Morabito, Giuseppa La Spada, Angela Marino, Silvia Dossena

**Affiliations:** 1 Institute of Pharmacology and Toxicology, Paracelsus Medical University, Strubergasse 21, A-5020 Salzburg, Austria; alessia.remigante@pmu.ac.at (A.R.); costa_roberta@hotmail.it (R.C.); 2 Department of Chemical, Biological, Pharmaceutical and Environmental Sciences, University of Messina, Viale F. Stagno D'Alcontres 31, I-98166 Messina, Italy; rmorabito@unime.it (R.M.); laspada.giuseppa@unime.it (G.L.S.); marinoa@unime.it (A.M.)

**Keywords:** Scyphozoa, nematocysts, toxin, venom, sting, first aid

## Abstract

Cnidaria include the most venomous animals of the world. Among Cnidaria, Scyphozoa (true jellyfish) are ubiquitous, abundant, and often come into accidental contact with humans and, therefore, represent a threat for public health and safety. The venom of Scyphozoa is a complex mixture of bioactive substances—including thermolabile enzymes such as phospholipases, metalloproteinases, and, possibly, pore-forming proteins—and is only partially characterized. Scyphozoan stings may lead to local and systemic reactions via toxic and immunological mechanisms; some of these reactions may represent a medical emergency. However, the adoption of safe and efficacious first aid measures for jellyfish stings is hampered by the diffusion of folk remedies, anecdotal reports, and lack of consensus in the scientific literature. Species-specific differences may hinder the identification of treatments that work for all stings. However, rinsing the sting site with vinegar (5% acetic acid) and the application of heat (hot pack/immersion in hot water) or lidocaine appear to be substantiated by evidence. Controlled clinical trials or reliable models of envenomation are warranted to confirm the efficacy and safety of these approaches and identify possible species-specific exceptions. Knowledge of the precise composition of Scyphozoa venom may open the way to molecule-oriented therapies in the future.

## 1. Introduction

Cnidarians (Hatschek, 1888) are one of the oldest phyla and have existed since at least the Cambrian [1]. These venomous animals are widespread in tropical and temperate oceans and seas, with very few freshwater species [2]. The consensus among taxonomists is that five classes should be included in this phylum: Anthozoa, Cubozoa, Hydrozoa, Scyphozoa, and Staurozoa [3,4,5]. Anthozoa are represented by sea anemones and corals and are sessile organisms in adult life. Conversely, in the last classes, which constitute the subphylum Medusozoa, the sexual form is generally represented by a free-living medusa, with the exception of Staurozoa, that are regarded as benthonic medusae [6].

**Figure 1:**
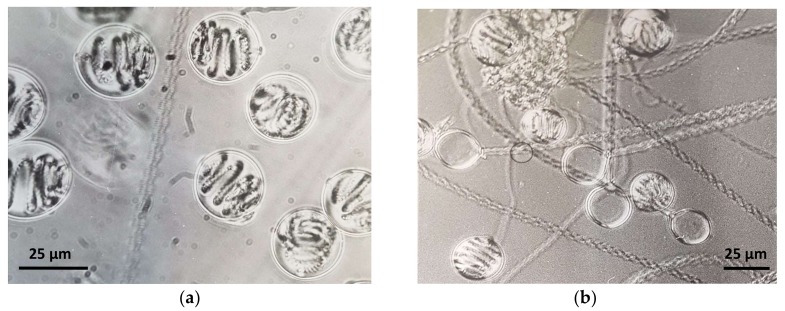
Phase contrast microphotographs of (**a**) undischarged and (**b**) discharged holotrichous-isorhiza nematocysts isolated from *Pelagia noctiluca* (Cnidaria: Scyphozoa) oral arms.

With more than 10,000 existing species, cnidarians are characterized by great biodiversity [7]. However, a characteristic common feature of these animals is the nematocyte, a cell type unique to this phylum. Nematocytes contain an extrusive organoid, the nematocyst (Figure 1), derived from the Golgi complex [6,8].

Nematocysts are used for prey capture, defense, spatial competition, and locomotion and may differ in size and shape between species and also within the same species. There have been 25 to 30 types described according to morphological properties [9,10]. Invariably, they present a common structure consisting of a cylindrical capsule closed by an operculum and containing an inverted tubule immersed in an aqueous solution with a complex mixture of toxins. Following mechanical–chemical stimulation, the tubule is quickly everted, thus injecting into the teguments of the prey or predator the venomous substances. This event is called discharge and is known as one of the fastest processes in the animal kingdom [8,11,12]. Recent studies reveal that a novel elastic protein, similar to that of spider silk, may be the molecular determinant of kinetic energy storage and release during nematocyst discharge [13].

Being present in both coastal and open waters at different depths, cnidarians often come into accidental contact with humans, interfering with human activities and consequently having a considerable impact on public safety, health, and economy. People engaged in recreational aquatic activities, such as swimmers, surfers and divers, might be exposed to cnidarian stings. With specific regard to Scyphozoa, abundant blooming of some species observed in the last decade in the Northeast Atlantic and the Mediterranean and in the coastal areas of Korea, China, and Japan have discouraged tourists and markedly interfered with fishing and aquaculture, thus causing substantial economic burden to coastal economies [14,15,16,17,18,19,20,21,22]. 

Concerning the impact of cnidarians on human health, the potentially lethal *Chironex fleckeri* (Cnidaria: Cubozoa), *Carukia barnesi* (Cnidaria: Cubozoa), and *Physalia* species (Cnidaria: Hydrozoa) are considered to be the most venomous among the Medusozoa [23,24,25]. Although the Scyphozoa are generally considered less dangerous, they are responsible for the majority of jellyfish envenomations throughout the world and life-threatening complications following this occurrence are possible [26]. The lay press, anecdotal reports, and various information accessible through the web concerning first aid measures for jellyfish stings are often confusing and contradictory. This may lead to the adoption of remedies that are ineffective or harmful and may delay or impede the application of more rationale measures of intervention. In addition, there is no consensus among the scientific reports on this subject, which is a matter of intense debate [27,28,29,30,31]. Therefore, the aim of the present work is to give an overview of the first aid measures for scyphozoan stings based on scientific information, with special emphasis on the mechanistic activity of specific venom components.

## 2. Components of Scyphozoan Venom and Their Activity

Among Medusozoa, only Scyphozoa are referred to as “true jellyfish” [32] and include approximately 200 extant species [4]. Regardless of the species, scyphozoan venom is a complex mixture of bioactive molecules. While substantial efforts have been devoted to the identification of the composition of Anthozoa venom, Scyphozoa venom is relatively less well characterized. The components of cnidarian venom exhibit striking diversity and include low molecular weight non-protein compounds such as serotonin and histamine, as well as high molecular weight complex proteins. This latter category includes enzymes, pore-forming toxins, and neurotoxins targeting voltage-dependent ion channels [12]. Of these, only enzymes (lipases and proteases) have been extensively characterized and firmly established as fundamental components of scyphozoan venom.

### 2.1. Lipases

#### 2.1.1. Phospholipase A2

Direct or indirect evidence of the presence of phospholipase A2 (PLA2) in Scyphozoa was first obtained from various tissue preparations. A polypeptide toxin with sequence similarity to PLA2 previously identified in other organisms was first isolated from the tentacles of the Mediterranean scyphozoan *Rhopilema nomadica* [33], a finding that was later confirmed by the detection of PLA2 catalytic activity in the venom of this species [34]. High PLA2 catalytic activity was also measured in tissue homogenates from *Cyanea capillata* (bell and tentacles) and *Aurelia aurita* [35]. Consistent with these findings, the concentration of malondialdehyde, a marker of lipid peroxidation, was increased in rat erythrocytes after treatment with a tentacle extract from *Cyanea capillata* [36], and two group 3 secretory PLA2 proteins were found in the nematocyst content of *Aurelia aurita* [37].

PLA2/PLA2-like catalytic activity has been detected in several fractions of venom from tentacle nematocysts of *Cassiopea xamachana* [38], cnidocyst extracts prepared from mesenteric and fishing tentacles of *Cyanea capillata* and *Cyanea lamarckii* [39], crude venom from nematocysts of *Cyanea nozakii* Kishinouye [40], extract of the tentacles of *Phyllorhiza punctata* [41], oral arms of *Rhopilema esculentum* [42], venom of *Chrysaora* sp. [43], and nematocyst venom of *Nemopilema nomurai* and *Cyanea nozakii* [44]. In *Stomolophus meleagris*, PLA2 was found to represent 21% of the venom proteins [45]. Transcripts of several isoforms of phospholipase PLA2 were found in *Cyanea capillata* tentacles [46]. Eight PLA2 family members were identified exclusively in the transcriptome—but not in the venom proteome—of *Chrysaora fuscescens* [47]. Finally, the presence of PLA2 was confirmed by mass spectrometry (MS) in the venom of *Cyanea nozakii* [48].

Secreted PLA2 has been found in several animal venoms [49] and probably contributes to the development of pain, inflammation, and cell lysis consequent to the envenomation [12].

#### 2.1.2. Phospholipase B, D, and Other Lipases

A single type B phospholipase (PLB2) and four lysosomal acid lipase (LAL)-like proteins were identified in the tentacle transcriptome but not in the venom proteome of *Chrysaora fuscescens* [47], while PLB1 was reported in *Stomolophus meleagris* [45]. The first report of type D phospholipase (PLD) in jellyfish species described five transcripts for PLDs in the tentacle of *Cyanea capillata,* which may explain the dermonecrotic activity of its venom [46]. Nematocyst venom proteome studies in *Nemopilema nomurai* and *Cyanea nozakii* disclosed the presence of PLD-like toxins at the protein level [44].

### 2.2. Proteases

#### 2.2.1. Metalloproteases

The first work suggesting the presence of metalloproteinases in scyphozoan venoms was published relatively recently [50]. *Nemopilema nomurai*, *Rhopilema esculentum*, *Cyanea nozakii*, and *Aurelia aurita* venoms showed gelatinolytic, caseinolytic, and fibrinolytic activities that were inhibited following treatment with 1,10-phenanthroline. Importantly, the metalloproteinase activity correlated with the cytotoxicity of venom. Later, the metalloproteinases found in the tentacle extracts of *Cyanea capillata* were suggested to be responsible for the hemorrhagic effects seen in the liver and kidney of animal models of delayed jellyfish envenomation syndrome [51].

Matrix metalloproteinase-14 and astacin-like metalloprotease were the main proteins found in the venom of *Nemopilema nomurai* by proteomic analysis [16], and metalloproteinases were assessed to represent 15% of the venom proteins of *Stomolophus melagris* [45]. Accordingly, multiple transcripts corresponding to various isoforms of matrix metalloproteinases and astacin-like metalloproteases were found in the tentacles of *Cyanea capillata* [46]. Metalloproteinase enzymatic activity was predominant in *Nemopilema nomurai* and *Cyanea nozakii* nematocyst venom [44] and could be attributed to zinc metalloproteinase-disintegrin-like and astacin-like metalloproteinases [52]. Accordingly, zinc metalloproteinase-disintegrin agkistin was found in the venom of *Cyanea nozakii* by MS analysis [48]. Eleven metalloproteinases—five of which are homologous to endothelin-converting enzyme (ECE) 1-like and 2-like proteins—have been identified in the venom proteome of *Chrysaora fuscescens* [47]. Zinc metalloproteinase nas-15-like was identified by proteomic analysis of whole body proteins of *Pelagia noctiluca* [53]. A 95 kDa metalloproteinase was partially purified from the tentacle extract of *Rhizostoma pulmo* [54].

Venom metalloproteases might participate in the process of digesting the prey through their proteolytic effects [52] or foster the penetration of other venom toxins into the target tissues [47].

#### 2.2.2. Serine Proteases

A factor that was inactivated following the exposure to a serine protease inhibitor, but void of proteolytical activity, was first identified in the content of fishing tentacle nematocysts of *Chrysaora quinquecirrha* [55]. Later, alpha-chymotrypsin-like serine protease activity was detected in the venom of *Rhopilema nomadica* nematocysts [34]. An enzyme with chymotryptic activity inhibited by the serine protease inhibitor phenylmethanesulfonyl fluoride was found in the nematocyst extract of *Nemopilema nomurai*. The amino acid sequence deduced from the corresponding cDNA sequence showed 41% identity with the human chymotrypsin-like (CTRL) and the CTRL-1 precursor [56]. The presence of fifteen unique transcripts belonging to the serine protease family of proteins was detected in the global transcriptome analysis of *Cyanea capillata* [46]. Serine proteases were found by MS in the venom of *Chrysaora fuscescens* [47], *Nemopilema nomurai*, and *Cyanea nozakii* [44].

The role of serine proteases in jellyfish venom is poorly understood; similar to what was observed in other venomous animals, these enzymes may play a role in the proteolytic permeabilization of the tissues of the prey and promote the penetration and/or activation of other venom proteins [46,57].

#### 2.2.3. Other Proteases

Other proteases identified in *Chrysaora fuscescens* that have not been identified in the venom proteomes of other jellyfish species include one cathepsin D-like protease, two aspartic peptidase-like proteases, and a predicted PC3-like endoprotease variant B isoform X1 [47].

### 2.3. Serine Protease Inhibitors

A screen of a cDNA library of *Cyanea capillata* identified one cDNA encoding a full-length serine protease inhibitor (Serpin), which was called jellypin. Jellypin exhibited serine protease inhibitory activity in vitro [58]. Later, the presence of multiple serine protease inhibitor transcripts was confirmed by a global transcriptome analysis of the tentacle of *Cyanea capillata* [46]. In this study, several serine proteinase inhibitors were identified, including Kazal-type, Kunitz-type, Papilin-like and Serpin B4. Representing as much as the 28% of venom proteins, serine protease inhibitors were the most abundant components of the venom of *Stomolophus meleagris* [45]. Kunitz-type proteins were also found in the nematocyst proteome of *Aurelia aurita* [37] and *Chrysaora fuscescens* [47].

Serpins are commonly found in the venoms of many animals [59,60] where they may protect toxin integrity. Establishing whether this is also the case in Scyphozoa deserves further investigation [46].

### 2.4. Hyaluronidases

The presence of hyaluronidases in the nematocyst venom of *Nemopilema nomurai* and *Cyanea nozakii* was recently disclosed by MS [44]. Hyaluronidases may play a role in the disruption of the extracellular matrix through the degradation of hyaluronic acid, thus facilitating the spread of toxins in the target tissues [61].

### 2.5. Deoxyribonucleases

It is surprising that these poorly characterized venom components were described relatively early in the venom of *Chrysaora quinquecirrha* [62]. Plancitoxin-like deoxyribonuclease transcripts were later found in *Cyanea capillata* and *Chrysaora fuscescens* [47]. 

Plancitoxins represent the lethal factors of the crown-of-thorns starfish *Acanthaster planci*, exhibit marked hepatotoxicity, amino acid sequence homology with mammalian deoxyribonucleases II (DNases II), and DNA degrading activity [63].

### 2.6. L-Amino Acid Oxidases

The presence of L-amino acid oxidases (LAAOs) was detected by MS in the nematocyst venom of *Nemopilema nomurai* and *Cyanea nozakii* [44].

LAAOs are flavoenzymes that catalyze the stereospecific oxidative deamination of an L-amino acid to produce the corresponding α-keto acid (2-oxo acid), ammonia, and hydrogen peroxide. LAAOs are particularly abundant in the venom of some species of snakes and contribute to the induction of envenomation effects, including modification of platelet aggregation, hemolysis and hemorrhage, stimulation of apoptosis, activation of leukocytes, and formation of edema [64]. 

### 2.7. C-Type Lectins

Transcripts of C-type lectins were detected in *Stomolophus meleagris* [45], *Aurelia aurita* [37], and *Chrysaora fuscescens* [47]. In *Stomolophus meleagris* and *Chrysaora fuscescens*, three C-type lectins were also found in the venom proteome [45,47].

C-type lectins are calcium-dependent carbohydrate-binding proteins that are ubiquitously expressed in nature and play a fundamental role in a variety of cellular processes where the phenomenon of recognition between a sugar moiety and a protein is involved [65]. C-type lectins are well-characterized components of the venom of many snakes and exhibit pro/anti-coagulant and pro/anti-thrombotic activities [66]. 

### 2.8. Neurotoxins and Other Ion Channel Blockers

Toxins targeting the voltage-gated (v) potassium (K+) and sodium (Na+) channels (neurotoxins) and other cation channels involved in pain transduction, such as the acid-sensing ion channel (ASIC) and the transient receptor potential cation channel subfamily V member 1 (TRPV1), are fundamental and well characterized components of venom of Anthozoa [12,67,68]. In particular, for these sessile organisms, neurotoxins are crucial in provoking paralysis of the prey or predator. However, information about the presence of neurotoxins and ion channel blockers in scyphozoan venom is sparse.

A novel low molecular weight (8.22 kDa) polypeptide with Na+(v) channel blocking activity was isolated from the fishing tentacle isorhiza nematocysts of *Cyanea capillata* [69].

Proteins defined as “K+ channels inhibitors” were assessed to represent 16% of venom proteins in *Stomolophus meleagris* [45]. A putative proteinaceous toxin comprising a ShKT domain, which is characteristic of a group of potent K+ channels blockers originally isolated from sea anemones, was found in the venom of *Chrysaora fuscescens* [47]. However, proteins comprising ShKT domains often bear additional functional modules and may, therefore, have hybrid functions in addition to or distinct from channel modulation; multiple transcripts and/or proteins of this type were found in *Chrysaora fuscescens* [47] and in *Aurelia aurita* [37].

One fraction of the tentacle venom extract from *Aurelia aurita* potently inhibited ACh-elicited currents mediated by both vertebrate fetal and adult muscle nicotinic acetylcholine receptor (nAChR) subtypes. The bioactive fraction contained a major protein component of ~90 kDa and was void of phospholipase A activity [70].

Altogether, the current knowledge on the venom composition of various cnidarian classes points toward the consideration that neurotoxin and ion channel blockers are not the main components of scyphozoan venom, though further studies are needed to better elucidate this point.

### 2.9. Pore-Forming Toxins

Pore-forming toxins appear to be a common component of cnidarian venoms and are particularly well characterized in Anthozoa [68] and Cubozoa [71]. Cnidarian pore-forming toxins are proteins of variable size and structure and can be broadly subdivided as follows [12,72]: (i) actinoporins (found in Anthozoa and Hydrozoa); (ii) jellyfish toxins (characteristic of Cubozoa); (iii) hydralysin-related toxins (found in Hydrozoa and Anthozoa); and (iv) membrane attack complex (MAC)-perforin (typically found in Anthozoa). Pore-forming toxins may very well explain the cytolytic and hemolytic activities of the venom of a number of cnidarian species, as assessed on a wide variety of biological substrates [72,73].

In Scyphozoa, pore-forming toxins are less well characterized. A cytotoxic protein (CcTX-1) was isolated from the fishing tentacle venom of *Cyanea capillata*. De novo sequencing of CcTX-1 revealed amino acid sequence similarity with known hemolytic proteins of two cubozoan species, i.e., CaTX-1 and CrTX-1 from *Carybdea alata* and *Carybdea rastonii*, respectively [74]. This and other findings led to the hypothesis that hemolysis induced by the venom of *Cyanea capillata* may be due to the formation of pores in the erythrocyte plasma membrane [36]. Five proteins similar to the CaTX family of hemolysins were also found in the venom of *Aurelia aurita*, together with as many MAC pore-forming putative toxins [37]. 

Similar to what was found for *Cyanea capillata*, the hemolytic potential of crude venom isolated from holotrichous-isorhiza nematocysts from the oral arms of *Pelagia noctiluca* was effectively inhibited by osmotic protectants but not by proteases or antioxidant compounds, thus leading to the hypothesis of a pore-forming mechanism rather than oxidative damage to the cell membrane [75]. These findings were corroborated by recent electrophysiological studies showing a large, amiloride-sensitive Na+ current in venom-treated cells due to a thermolabile venom component. Interestingly, the ion current appeared following application of venom from both the extracellular and intracellular sides of the plasma membrane, a behavior that is more compatible with the insertion of a porin-like component than activation of endogenous ion channels. Importantly, removal of NaCl from the bath solution significantly blunted the venom-induced current and cell swelling [76].

Two putative pore-forming toxins (PFTs) were identified in the venom proteome of *Chrysaora fuscescens*. One of these potential toxins (CfusTX-1) shares high sequence similarity with predicted toxins TX1 and TX2 from *Aurelia aurita* and other cubozoan toxins, while the other is a novel protein [47]. A transcript with significant similarity to TX2 of *Aurelia aurita* was also found in the tentacle transcriptome of *Cyanea capillata* [46].

### 2.10. Cysteine-Rich Secretory Proteins

Six venom allergen-like proteins of the cysteine-rich secretory protein (CRISP), allergen V5/Tpx-1-related protein family, were identified in the *Chrisaora fuscescens* venom proteome [47]. While already reported in the venom of box jellyfish [77] and commonly found in the venom of certain snakes and lizards [78], the role of CRISP proteins in Scyphozoa remains uncertain.

### 2.11. Other Venom Components of a Protein Nature

The detection of ATPase-like (Av120) proteins (*Aurelia aurita*; [37]), a glycoside hydrolase protein (*Chrysaora fuscescens*; [47]), and various transcripts showing similarity to histamine-releasing factor (HRF), angiotensin-converting enzyme-like (ACE-like) proteins, endothelin-converting enzyme 1-like (ECE 1-like) proteins, vascular endothelial growth factors, lysosomal acid lipases (LALs), alkaline phosphatase, dipeptidyl peptidase 3, and ectonucleoside triphosphate diphosphohydrolase (*Cyanea capillata*; [46]) in the venom proteome and transcriptome of some Scyphozoa highlights the complex composition of the venom of these animals, which is only partially understood.

## 3. Reactions to Scyphozoan Stings

Cnidaria include the most venomous animals of the world. Fatalities consequent to cnidarian stings may result from the action of toxins on vital organs such as the heart, respiratory center, or kidneys, as well as—although rarely—from anaphylaxis [79]. *Chironex fleckeri* antivenom is the only jellyfish antivenom available [80], has been in use since 1970, and is useful also in the treatment of *Chiropsalmus sp*. stings [24,25,81]. The most effective therapy for Irukandji syndrome, which is due to *Carukia barnesi* stings, seems to be an intravenous infusion of magnesium [25]; pain relief can be obtained with opioids [82]. The majority of cnidarian stings lack lethal potential. However, the severity of local and systemic reactions depends on the specific composition of the venom of a given species or specimen, the volume of venom injected, the area and duration of contact between the skin of the victim and the tentacle, and the general health status and age of the victim [12].

Although Scyphozoa are considered relatively less harmful compared to Cubozoa and other cnidarians and life-threatening complications following scyphozoan stings are rare, these animals are still capable of inducing local and systemic reactions deserving medical attention.

### 3.1. Local Reactions to Scyphozoan Stings

Immediate reactions to scyphozoan stings include a linear vesiculourticarial eruption at the site of contact, possibly with a hemorrhagic or necrotic component, evolving into erythema and/or edema and associated with itching and/or stinging pain of various intensity depending upon the species of the jellyfish [81]. Dermoscopic findings may be species-specific and represent a diagnostic tool of jellyfish sting when a clear history of contact is lacking [83]. Although pain and erythema usually resolve in a timeframe varying from hours to days, sequelae of the primary rash may include hyperpigmentation and scaring. The pathogenesis of these acute inflammatory lesions is due to the direct toxic effect of jellyfish venom and normally the reaction occurs immediately after the sting. Management usually includes oral or topical antihistamines and topical corticosteroids. In severe cases, systemic corticosteroids may be needed. Analgesics (acetaminophen, non-steroidal anti-inflammatory drugs such as ibuprofen, and opiates) and topical antibiotics may help control the pain and prevent or treat infections, respectively. Antibiotics should cover *Staphylococcus*, *Streptococcus*, and microbes of marine origin, such as *Vibrio* [84,85,86,87,88]. Ocular stings may represent an ophthalmic emergency and have been managed successfully with topical cycloplegics, topical steroids, topical antibiotics, and topical antihistamines [89].

Cutaneous eruptions may become generalized or persistent and their appearance can be delayed in time or distant from the site of the primary sting [79]. Delayed reactions can be severe and have been observed following contact with *Rhopilema nomadica* and *Rhizostoma sp*. [90,91]. Recurrent reactions at the same anatomic site of the primary sting seen several days after resolution of the initial rash can also occur [92]. Species involved in this phenomenon include *Pelagia noctiluca*, and probably *Lychnorhiza lucerna*, *Rhizostoma pulmo*, or *Aurelia aurita* [93]. Recurrent reactions indicate activation of the immune system by venom components and/or a prolonged residency of the toxin in the tissues of the victim [25]. The histology of recurrent cutaneous eruptions usually shows significant lymphocytic perivascular inflammatory infiltrates with eosinophils. These histologic findings may be due to a type IV allergic reaction elicited by Langerhans cells and helper T-lymphocytes. Topical immunomodulators (tacrolimus) and intralesional injections of steroids may help to resolve these lesions [94].

### 3.2. Systemic Reactions

Theoretically, a venom component spreading in the systemic circulation may elicit systemic toxic reactions with injury to various organs, as well as allergic reactions. Although less frequent and severe than those observed with some Cubozoa and Hydrozoa species, systemic reactions after contact with some Scyphozoa specimens are possible and are reviewed briefly in the following, based on the scyphozoan species.

*Stomolophus meleagris*, also called cannonball jellyfish or cabbage head jellyfish, is particularly abundant along the southeastern coast of the United States and East Central Pacific Ocean. In China, it is considered one of the most dangerous jellyfish. In addition to local symptoms, including itching and edema, victims may present with systemic symptoms, such as myalgia, dyspnea, hypotension, shock, and death [45].

*Cyanea capillata*, also known as the lion’s mane jellyfish, giant jellyfish or the hair jelly, is a large jellyfish that can be found in temperate, boreal, and polar regions of the Atlantic and Pacific Oceans [95]. Encounters with this specimen may become problematic due to its large size and the considerable number and length of its thin tentacles. Systemic symptoms reported after contact with this jellyfish include nausea, sweating, dizziness, and abdominal and muscular cramps [96]. In very severe stings, difficulties in breathing, pain on respiration, tachycardia, muscle spasms, stiffness of back and joints, and the development of an Irukandji-like syndrome have been reported [95]. As previously mentioned, symptoms of Irukandji syndrome are classically reported as a possible consequence of envenomation from *Carukia barnesi* and various species of the Carybdeida order.

*Pelagia noctiluca*, also referred to as “the mauve stinger”, is a ubiquitous species, but is commonly found in the Mediterranean Sea where it is considered the most venomous autochthonous jellyfish [97]. Although very painful, stings inflicted by these specimens are generally not life threatening. However, generalized allergic reactions such as bronchospasm and dyspnea are possible; one case was reported of anaphylaxis with hypotension and bronchospasm following exposure to a *Chrysaora* toxin as a consequence of a previously developed sensitization towards a *Pelagia* antigen conserved in *Chrysaora* [98]. A case of Guillain-Barré syndrome was also described. The victim described tingling in both heels that spread to the hands, weakness of the limbs, unsteady gait, diminished sensation of touch, and an absence of tendon reflex. The diagnosis was obtained by nerve conduction studies showing prominent demyelinating neuropathy with conduction block, and an aberrant immune response to a toxic venom component was postulated [99].

*Rhopilema nomadica*, also called “the nomad jellyfish”, is indigenous to the Indian and Pacific Oceans. However, in the 1970s, it invaded the Mediterranean Sea via the Suez Canal and started to proliferate considerably [100]. Possible dangerous situations linked to this specimen are caused by its large aggregations and its involvement with the pediatric population. A severe case of anaphylactic reaction as a direct consequence of a *Rhopilema nomadica* sting was reported in 2016 and is the first report of anaphylaxis due to a Mediterranean jellyfish envenomation. The patient presented with difficulties in breathing, hoarseness, itching, periorbital swelling, and facial edema and required hospitalization [101]. A recent retrospective study described systemic symptoms in children including fever, chills, tachycardia, muscle spasms, severe cellulitis, and two cases of anaphylactic reaction [87].

*Chrysaora sp*. is widely distributed in the Atlantic, Pacific, and Indian Oceans and is commonly named “sea nettle”. Persistent urinary incontinence and biliary dyskinesia were described following a serious sting by *Chrysaora fuscescens* on the abdomen. The gallbladder disorder required surgery and the urinary bladder dysfunction resolved spontaneously over 20 months [102].

*Aurelia aurita*, a species abundant in the Mediterranean Sea, may cause, in addition to dermatonecrosis, systemic symptoms such as fever, dyspnea, and muscle weakness [103].

## 4. First Aid Measures for Scyphozoan Stings

Common sense dictates that prevention of jellyfish stings rather than treatment would be the better option. Beach closures and suspension of aquatic events in the case of jellyfish swarms, on-site protective nets in the water, and warning signs on beaches would work in this sense. These measures have been applied at least partly for *Cyanea capillata* and *Pelagia noctiluca* among Scyphozoa [95,104], and have also been put in place in Australia for the more dangerous Cubozoa, including the deadly *Chironex fleckeri* [105], and are advisable elsewhere [106].

In case of scyphozoan stings, as in the case of other marine envenomations, management first necessitates rescue and prevention of drowning and close monitoring of systemic reactions, especially anaphylaxis, which would require basic and advanced life support measures [104]. If this is not the case, first aid measures can be initiated with the aim of (i) preventing further discharge of nematocysts and (ii) limiting the action of the venom in terms of pain and tissue damage [28,96].

### 4.1. Prevention of Further Discharge of Nematocysts After a Sting

Preventing further nematocyst discharge after a sting would limit the venom load and implies (i) removal of the tentacles and (ii) inactivation of nematocysts that may be adhering to the skin.

Tentacle removal from the skin of the victim is necessary as detached tentacles are still capable of envenomation. Tentacle removal is a critical step as the procedure may trigger further nematocyst discharge. However, very few studies focus on this issue, especially concerning Scyphozoa [28]. At best, tentacles should be removed with tweezers or similar tools as using bare hands may cause injury to the rescuer [96]. Scraping of the sting site with various objects, such as a credit card or a razor, was proven deleterious in the case of Cubozoa stings [30]. As the application of pressure during this procedure may produce further nematocyst discharge, it is likely that it should also be avoided in the case of scyphozoan stings. For the same reason, pressure-inducing bandages (PIB) are no longer recommended [31]. While it has been proposed to cover the stung body part with sand to enclose tentacle remnants and ease their removal, this practice seems to lack evidence and consensus [107,108].

#### 4.1.1. Rinsing with Seawater

Tentacle removal can also be attempted by thoroughly washing the area with an aqueous solution. Care should be taken in choosing a washing medium that does not exacerbate pain or induce further nematocyst discharge. Indeed, the optimal washing medium should rinse off tentacles while inactivating nematocysts. However, the adoption of efficient and safe rinsing methods is hampered by a lack of consensus between recommendations or absence of evidence-based support. In addition, in the medical literature, as well as in the lay information accessible via the internet, recommendations are often given generally and refer to all cnidarians, or all “jellyfish”, with lack of a species-specific approach. For example, rinsing the sting site with seawater was found to help in tentacle removal, relief of pain, and inhibition of venom discharge [96] and is generally recommended in the case of *C*. *capillata* stings [31,96,109,110,111] and for *A. aurita*, *N. nomurai*, and *P. noctiluca* stings (Table 1). However, a very recent study based on a combination of in vitro nematocyst discharge tests and envenomation models concluded that seawater rinsing induced significant increases in venom delivery and, therefore, its use for *C. capillata* stings should be discontinued [95]. Given that seawater rinsing was found to increase nematocyst discharge also in *P. noctiluca* (Table 1), further studies are needed to evaluate the efficacy of this procedure in the case of stings from scyphozoan species. Other studies have found that NaCl induces nematocyst discharge (Table 1) and the ion current induced by the crude venom of *P. noctiluca* is suppressed in a NaCl-free solution [76], supporting the dismissal of seawater (which is notably rich in NaCl) as a rinsing solution.

#### 4.1.2. Rinsing with Vinegar or Acetic Acid

Another issue that lacks consensus in the scientific literature is the efficacy of rinsing the sting site with vinegar. Vinegar was contraindicated for *C. capillata* stings and, in general, for scyphozoan stings including *Aurelia aurita*, *C. quinquecirrha*, *N. nomurai*, and *P. noctiluca* (Table 1) as it was reported to either increase venom load or exacerbate pain [96]. However, a more recent study showed that vinegar, although inducing cnidae discharge in vitro, significantly decreased venom functional activity in *C. capillata* [95]. Similarly, a previous study demonstrated that 5% acetic acid did not induce nematocyst discharge *per se* and dramatically impaired discharge induced by a combined physico-chemical stimulation of oral arms of *P. noctiluca* [121]. This evidence strongly suggests that recommendations against the use of domestic vinegar (4–6% acetic acid) or diluted acetic acid as rinsing solutions after scyphozoan stings should be carefully re-evaluated.

#### 4.1.3. Rinsing with Deionized, Distilled, or Fresh Water

Generally, it is recommended to avoid rinsing with fresh water in the case of a jellyfish sting as this procedure may induce nematocyst discharge [96] by osmotic challenge and did not appear to produce any noticeable improvement in pain sensation [28]. However, most reports refer to Cubozoa. With specific regard to Scyphozoa, deionized water was found to be ineffective for *C. quinquecirrha* (Table 1). Distilled water did not prevent nematocyst discharge in *P. noctiluca* [123]. Other authors have strongly recommended avoiding freshwater as the osmotic or pressure changes may trigger discharge of unfired nematocysts [31]. Overall, there is no evidence in favor of the use of plain water at ambient temperature for scyphozoan stings.

#### 4.1.4. Rinsing with Urine or Solutions Containing Urea or Ammonia

Urine is a well-known folk remedy for jellyfish stings [31]. This led to an investigation of the possible use of urea—which is notably the largest solid constituent of human urine—and ammonia in the first treatment of cnidarian stings. However, urine was soon recognized to be of no help [24] or even detrimental because, for example, it led to a dramatic increase in nematocyst discharge in the Cubozoan *Alatina alata* [127]. Concerning Scyphozoa, urine, urea, and ammonia were found to be either detrimental or ineffective with the notable exception of *P. noctiluca* where ammonia was found to inhibit the in situ chemosensitizer-induced nematocyst discharge (Table 1). These findings may indicate that species-specific responses to a given chemical or treatment are possible. In addition, differences among the various models of investigation have to be taken into account. Despite these discrepancies in the scientific literature, ammonia continues to be used as the main (74%) non-pharmacological treatment for jellyfish stings along the south Italian coast where most stings (61.81%) are attributed to *Pelagia noctiluca* [125]. We believe that assessing whether ammonia can be used safely and efficaciously in the first treatment of patients with *P. noctiluca* stings is worth further investigation. 

#### 4.1.5. Additional Rinsing Solutions

Among the various rinsing solutions that were assayed (acetone, alcohols including 70% ethanol and methylated spirits, and alkaline solutions), only methylated spirits were found to be of some benefit for scyphozoan stings (Table 1). Again, while generally found to be detrimental, 70% ethanol inhibited the *in situ* chemosensitizer-induced nematocyst discharge in *P. noctiluca* (Table 1). Stingose, an aqueous solution of 20% aluminum sulfate and 1.1% surfactant [128], was found to be of benefit for *C. quinquecirrha* and *C. capillata* stings as it inhibited nematocyst discharge (Table 1). Commercially available proprietary formulations, such as the newly-developed Sting No More^®^ Spray (contents include vinegar, copper gluconate, urea, and magnesium sulfate), formerly found useful in the case of Cubozoa stings [127], also reduced venom load in an *ex vivo* model of envenomation from the scyphozoan *C. capillata* [95], and may work well for other scyphozoan stings.

#### 4.1.6. Bicarbonate and Baking Soda Slurry

A baking soda slurry was found to prevent *P. physalis* (Hydrozoa) nematocyst rupture in vitro [112] and inactivate *C. rastoni* (Cubozoa) nematocysts [129] with an overall positive effect in the treatment of European jellyfish species [31]. Concerning Scyphozoa (Table 1), baking soda slurry was of potential benefit following *C. quinquecirrha* and *C. capillata* stings. There is no consensus for *P. noctiluca*. Thus, this remedy may be of help and further studies are needed to confirm species-specific differences in its efficacy.

### 4.2. Limiting the Action of the Venom in Terms of Pain and Tissue Damage

Again, there is no consensus on the action that should follow tentacle removal from the stung epidermis. The main objectives at this stage are to attenuate local pain and inflammation while preventing tissue damage, inactivating venom toxins, and preventing venom diffusion and discharge of unfired nematocysts that can still adhere to the epidermis. The resources immediately available *in loco* can be limited.

#### 4.2.1. Heat or Cold Packs

Considerable effort and debate have been devoted in establishing whether exposing the injured body part to cold or heat would be the best first aid treatment for jellyfish stings [29]. Cold packs, ice, or immersion in cold water would limit inflammation and diffusion of the venom and relieve pain, and indeed have been formerly recommended for Hydrozoa [117] and Cubozoa [130]. However, a relatively recent Cochrane Review of randomized controlled clinical trials focusing on *Physalia*, *Carukia*, and *Carybdea* sp. did not find sufficient evidence in favor of a specific treatment. While hot water immersion induced clinically significant pain relief, no statistically significant differences between hot water immersion and hot packs were found for the dermatological outcomes [131]. A more recent systematic review including all Cnidaria concluded that “the majority of studies to date support the use of hot-water immersion for pain relief”, and indicated that a 45 °C water immersion of the stung part for 20 min was the best treatment [29]. This conclusion is well supported by common knowledge that the venom of several animals—including Cnidaria—is thermolabile. However, it was argued that rather than denaturing venom components, heat would interfere with the pain transmission from peripheral nociceptors to central neurons, thus “opening the pain gate” circuit and relieving the pain [132]. The same effect would be obtained by counter-irritation of the sting site by rubbing sand on the injured part after all tentacle material has been removed [133]. However, other authors have recommended avoiding abrasive activity [134].

With specific regard to Scyphozoa (Table 1), heat was unequivocally found to be beneficial for *A. aurita*, *R. nomadica*, and *S. meleagris* stings; for *C. quinquecirrha*, a species possessing a relatively thermostable venom [135], both heat and cold exposure were ineffective. Conversely, for *C. capillata* and *P. noctiluca*, both cold and hot packs have been found to be of benefit in different studies (Table 1). Specifically, ice pack application was found useful in relieving the pain of stings from both species. However, utilizing an *ex vivo* model of envenomation, Doyle et al. found that heat inhibited the *C. capillata* venom activity while cold exposure was ineffective. As the model used in their study did not include metrics for pain or neurological processes, these authors were able to affirm that heat has a direct effect on venom proteins rather than an indirect, modulating effect on pain sensory systems, and recommended a 45 °C water immersion for 40 min as the best treatment for *C. capillata* stings [95]. These findings are in agreement with recent investigations indicating that the current-inducing active component of *P. noctiluca* venom is thermolabile [76]. Further studies are needed to better define the lowest temperature leading to venom inactivation in *P. noctiluca*.

#### 4.2.2. Lidocaine and Local Anesthetics

Once again, discordant opinions are found in the scientific literature. Topical anesthetics, such as lidocaine, have been found to be ineffective unless applied with an occluding bandage, and are slow acting and even toxic if applied to a large body area [88]. Conversely, a relatively recent systematic review of various treatments for envenomation by jellyfish and related organisms in North America and Hawaii identified topical lidocaine, as well as hot water, as one of the best systems to reduce pain [28]. Specifically regarding Scyphozoa (Table 1), local anesthetics were found to decrease skin redness and relieve pain after *C. quinquecirrha* stings. The onset in time of pain relief was dependent on the lidocaine concentration, with 10% and 15% lidocaine producing immediate relief [113]. These findings would be in agreement with the observations of an inhibition of *in situ* chemosensitizer-induced nematocyst discharge with 1% lidocaine in *P. noctiluca* [121]. At present, there is little evidence against the use of lidocaine, although it may be rarely available on the scene. Controlled clinical trials are warranted to unequivocally assess safety and efficacy of the use of lidocaine and other topical anesthetics after scyphozoan stings.

## 5. Further Considerations and Hints for Future Studies

Additional remedies, such as papain, have been found to be of benefit for the stings of some species (Table 1 and [136]), but can hardly be found *in loco*.

Melatonin (*N*-acetyl-5-methoxytryptamine), an efficient free-radical scavenger and antioxidant with possible immunomodulatory activity, reduced the acute inflammatory response elicited by *P. noctiluca* crude venom injection in the rat paw, and was therefore proposed as a potential novel treatment of local acute inflammation, including inflammation induced by *P. noctiluca* stings [137]. In this study, melatonin was administered intraperitoneally to rats. What the best route of administration of melatonin would be to treat local acute inflammation (systemic or transdermal [138]) in humans is currently not known.

With the increasing knowledge gathered by MS on the protein composition of jellyfish venoms, attempts are being made to identify molecule-oriented therapeutic approaches. For example, based on the finding that metalloproteinases are the central toxic component of the jellyfish *Cyanea capillata* tentacle extract, Batimastat (BB-94), a potent, broad-spectrum matrix metalloprotease inhibitor, attenuated multiple organ hemorrhagic injuries in a mouse model of delayed jellyfish envenomation syndrome [51]. Similarly, tetracycline (an antibiotic with matrix metalloproteinase inhibitory activity) significantly reduced progression of dermal toxicity upon inoculation of *Nemopilema nomurai* venom into rabbit skin [139]. Batimastat and Varespladib, a potent and selective human non-pancreatic secretory phospholipase A2 inhibitor, significantly reduced the hemolysis induced by *Nemopilema nomurai* venom [52]. These findings indicate that matrix metalloproteases and PLA2s and possibly other PLs can be regarded as novel therapeutic targets in scyphozoan envenomation. Further studies are needed on this subject. 

## 6. Conclusions

To conclude, we envision that first aid protocols for scyphozoan stings should:

- Recommend avoiding urine and distilled or plain water for rinsing the sting site;
- Consider dismissing seawater for rinsing the sting site;
- Recommend using vinegar/5% acetic acid for rinsing the sting site, especially for *C. capillata* and *P. noctiluca* stings;
- Include treating the sting site with hot packs or lidocaine.

Ammonia and baking soda slurry can be of benefit but most likely not for all species; a baking soda slurry can be useful for *C. quinquecirrha* and *C. capillata* stings. Further studies are needed to support the use of ammonia and clarify the usefulness of a baking soda slurry for *P. noctiluca* stings.

Avoiding useless or noxious procedures (such as rinsing the sting site with urine, seawater, or plain water) would represent a first achievement. However, some first aid measures that are clearly recognized as effective (such as vinegar and hot packs) are not readily available on-site. We propose that vinegar and hot packs should be made accessible at tourist beaches, especially during jellyfish blooming periods. Educating the personnel who may be involved in offering first aid assistance (lifeguards, medical first aid staff) is needed. Surveillance and informing the public on local jellyfish occurrence and species composition will be crucial in preventing and treating jellyfish stings.

## Figures and Tables

**Table 1:** First aid topical treatments for scyphozoan stings.

Species	Remedy	Effect	Mechanism	References
*Aurelia aurita*	Heat	Beneficial	Venom inactivation	[31,110]
	Seawater	Beneficial	Removing tentacles	[31,110]
	Vinegar	Deleterious	Pain exacerbation	[31,110]
*Chrysaora quinquecirrha*	Acetone	Deleterious	Nematocyst discharge	[96,112]
	Ammonia (20%)	Deleterious	Nematocyst discharge; Pain exacerbation	[96,112,113]
	Bromelain (10%)	Deleterious	Nematocyst discharge; Ineffective on pain	[96,113]
	Deionized water	Ineffective	Ineffective on pain	[113]
	Ethanol (70%)	Deleterious	Nematocyst discharge; Pain exacerbation	[96,113]
	Hot water	Ineffective	-	[96,114]
	Ice pack	Ineffective	-	[96,114]
	Lidocaine (5–15%)	Beneficial	Reduced pain, swelling, redness	[96,113]
	Meat tenderizer	Beneficial	Inhibited nematocyst discharge	[112]
	NaHCO_3_/baking soda slurry	Beneficial	Inhibited nematocyst discharge	[88,96,112,115]
	NaClO	Deleterious	Nematocyst discharge	[96,112]
	Papain	Beneficial	Inhibited nematocyst discharge	[96,112,116]
	Seawater	Deleterious	Induced nematocyst discharge	[112]
		Ineffective	Ineffective on pain	[96,113]
	Stingose	Beneficial	Inhibited nematocyst discharge	[96,112]
	Urea (10%)	Ineffective	Ineffective on pain	[96,113]
	Vinegar (5% acetic acid)	Deleterious	Nematocysts discharge; Pain exacerbation	[96,112,113]
*Cyanea capillata*	Acid	Deleterious	Nematocyst discharge	[31,96,111]
	Alcohol	Deleterious	Nematocyst discharge	[31,96,111]
	Cold/ice pack	Beneficial	Relieved pain	[24,31,96,117]
		Ineffective	-	[95]
	Heat/hot pack	Beneficial	Reduced venom activity	[31,95,110]
	Isopropanol	Deleterious	Nematocyst discharge	[95]
	Methylated spirits	Beneficial	Did not induce nematocyst discharge	[96,111]
	NaHCO_3_/baking soda slurry	Beneficial	Inhibited nematocyst discharge	[31,88,96]
	Seawater	Beneficial	Removing tentacles; Did not induce nematocyst discharge	[31,96,109,110,111]
		Deleterious	Increased venom load	[95]
	Sting No More^®^ Spray	Beneficial	Reduced venom activity	[95]
	Stingose	Beneficial		[31]
	Urine/Urea	Deleterious	Nematocyst discharge	[31,96,111]
	Vinegar	Deleterious	Nematocyst discharge; Pain exacerbation	[24,31,110,111,118]
		Beneficial	Reduced venom load	[95]
*Nemopilema nomurai*	Ethanol (70%)	Deleterious	Nematocyst discharge	[119]
	Isopropanol	Deleterious	Nematocyst discharge	[119]
	Seawater	Beneficial	Ameliorated pain and redness; Did not induce nematocyst discharge	[119]
	Vinegar (4% acetic acid)	Deleterious	Nematocyst discharge	[119]
*Pelagia noctiluca*	Acidic pH (4.5–6.5)	Beneficial	Inhibited nematocyst discharge	[120]
		Deleterious		[31]
	Alcohol	Deleterious		[31]
	Ammonia (20%)	Beneficial	Inhibited nematocyst discharge	[31,121]
	NaCl	Deleterious	Nematocyst discharge	[96,122]
	NaHCO_3_/Baking soda slurry	Deleterious		[31]
	Bases	Deleterious		[31]
	Cold/ice pack	Beneficial	Pain relief	[24,31,97]
	Distilled water	Ineffective	Did not affect nematocyst discharge	[123]
	Ethanol (70%)	Beneficial	Inhibited nematocyst discharge	[121]
	Heat/hot pack	Ineffective		[31]
		Beneficial	Inactivated venom	[76,110,124]
	Lidocaine (1%)	Beneficial	Inhibited nematocyst discharge	[121]
	Methylated spirits	Beneficial	Inhibited nematocyst discharge	[97]
	Seawater	Deleterious	Nematocyst discharge	[97,125]
		Beneficial	Removing tentacles	[31,110]
	Vinegar (5% acetic acid)	Deleterious	Nematocyst discharge; Pain exacerbation	[24,31,97,110,111]
		Beneficial	Inhibited nematocyst discharge	[121]
*Rhopilema nomadica*	Heat	Beneficial		[31]
	Vinegar	Ineffective	Did not alleviate pain	[90]
*Stomolophus meleagris*	Heat	Beneficial	Toxin inactivation	[126]

## Abbreviations

PL	phospholipase
MS	mass spectrometry
